# Examining the Financial Feasibility of Using a New Special Health Fund to Provide Universal Coverage for a Basic Maternal and Child Health Benefit Package in Nigeria

**DOI:** 10.3389/fpubh.2018.00200

**Published:** 2018-07-23

**Authors:** Obinna Onwujekwe, Chima Onoka, Ifeoma Nwakoby, Hyacinth Ichoku, Benjamin Uzochukwu, Hong Wang

**Affiliations:** ^1^Health Policy Research Group, Department of Pharmacology and Therapeutics, College of Medicine, University of Nigeria Enugu-Campus, Enugu, Nigeria; ^2^Department of Health Administration and Management, College of Medicine, University of Nigeria Enugu-Campus, Enugu, Nigeria; ^3^Department of Community Medicine, College of Medicine, University of Nigeria Enugu-Campus, Enugu, Nigeria; ^4^Department of Banking and Finance, University of Nigeria Enugu-Campus, Enugu, Nigeria; ^5^Department of Economics, University of Nigeria, Nsukka, Nigeria; ^6^Bill and Melinda Gates Foundation, Seattle, WA, United States

**Keywords:** financial feasibility, MCH, health fund, financing, Nigeria, universal health coverage

## Abstract

**Background:** A special health fund was established in Nigeria in 2014 and is known as the Basic Health Care Provision Fund (BHCPF). The fund is equivalent to at least 1% of the Consolidated Revenue of the Federation. The BHCPF will provide additional revenue to fund primary healthcare services and help Nigeria to achieve universal health coverage (UHC). This fund is to be matched with counterpart funds from states and local government areas (LGAs), and is expected to provide at least a basic benefit health package that will cover maternal and child health (MCH) services for pregnant women and under-five children.

**Objective:** To determine the financial feasibility of using the BHCPF to provide a minimum benefit package to cover all pregnant women and under-five children in Nigeria.

**Methods:** The study focused on three states in Nigeria: Imo, Kaduna, and Niger. The feasibility analysis was performed using 3 scenarios but the main analysis was Scenario 1, which was based on the funding of drugs and consumables only. All the costs and revenues were in 2015 levels. The standard costs of a minimum benefit package for the different states were multiplied by the number of target beneficiaries to determine the amount required for the year. Financial feasibility is determined by the excess or otherwise of revenue over costs.

**Findings:** It was found that in the best case funding scenario of using 95% of the CRF with 25% counterpart funding from states and LGAs, the entire available funds were not adequate to cover the benefit package for all the pregnant women and under-five children in the three states. The funds were also inadequate to cover the target beneficiaries that live below the poverty line in two of the states.

**Conclusion:** The BHCPF is a good step toward providing essential MCH services, but the current level of funding will not assure UHC for all the target beneficiaries. However, the available funds should be used immediately to target priority mothers and children such as vulnerable groups, whilst sourcing for additional funds to ensure universal coverage of MCH services.

## Introduction

Nigeria has a maternal mortality ratio of 576 per 10,000 live birth and Infant and under-5 mortality rates of 69 and 128 deaths per 1,000 live births, respectively (1). At these mortality levels, one in every 15 Nigerian children dies before reaching age one, and one in every eight does not survive to his/her fifth birthday. The lifetime risk of maternal death indicates that 1 in 30 women in Nigeria will have a death related to pregnancy or childbearing (1). Although many of these deaths are preventable, problems with access to health facility, attendance by skilled birth personnel and coverage and quality of health care services contribute to the elevated morbidities and mortalities. Much of these challenges arise due to inadequate and ineffective funding of maternal and child health interventions.

The defunct Millennium Development Goals (MDG) office in the Nigerian presidency, using funds from the Debt Relief Gains (DRG), funded the National Health Insurance Scheme (NHIS) to implement a free Maternal and under-five children's healthcare program in some states in the country. The program, which was titled the “NHIS-MDG Maternal and Child Health Project,” was implemented between 2008 and 2015. The project was an exemption scheme for Maternal and under-five Child Health services at the primary healthcare level. The participating states were expected to provide counterpart funding to expand coverage. The benefit package of the Free MCH program included primary health services for children <5 years, basic antenatal services and secondary care for pregnant women (2). An estimated 1,100,000 lives (including women and children) were expected to be provided with this service. The project was implemented in 12 states over two phases as follows: Phase I: Bayelsa, Gombe, Imo, Niger, Oyo & Sokoto, and Phase II: Bauchi, Cross River, Jigawa, Katsina, Ondo, and Yobe.

Although there is no hard evidence from an impact evaluation, anecdotal evidence shows that the Free MCH program provided needed services to so many women and children. It also led to improvements in infrastructure and quality of healthcare services in the participating states. Hence, there is a clamor from both beneficiaries, providers and other health system stakeholders for the reactivation and scaling-up of the program either in the original or in a new form. The passage of the National Health Act in 2014 and the legal creation of a Basic Health Care Provision Fund (BHCPF) in the Act thus provide an opportunity to achieve this.

The possibility to reactivate and scale-up the Free MCH program has been provided with the passage of the National Health Act in 2014 of Nigeria and the legal creation of a Basic Health Care Provision Fund (BHCPF) in the Act. The BHCPF is equivalent to at least 1% of the Consolidated Revenue Fund (CRF) of the Federation and it will be used to provide an essential package of health services to citizens, and to improve the infrastructure, drugs and consumables in primary healthcare facilities in Nigeria. This fund is to be matched with counterpart funds from states and local governments, and will be allocated through the National Health Insurance Scheme (NHIS [50%]), the National Primary Health Care Development Agency (NPHCDA [45%]) and the Federal Ministry of Health (FMOH [5%]). Hence, the BHCPF will provide additional revenue to support primary healthcare services and achievement of universal health coverage in the country when appropriated and released.

However, a financial feasibility analysis is required to provide needed information on how viable the fund will be in providing a thin or basic benefit health package of MCH services. The World Health Organization Centre for Health Development (3) defines financial feasibility as, “The projected ability of a provider to pay the capital and operating costs associated with the delivery of a proposed service.” Financial feasibility analysis is thus an analytical tool used to evaluate the economic viability of an investment (4). It consists of evaluating the financial condition and operating performance of the investment and forecasting its future condition and performance (4). There are many reasons for undertaking financial feasibility and they include: gives focus to the project and outline alternatives; Narrows business alternatives; Identifies new opportunities through the investigative process; Identifies reasons not to proceed with the project; Enhances the probability of success by addressing and mitigating factors early on that could affect the project; Provides quality information for decision making; Provides documentation that the business venture was thoroughly investigated; Helps in securing funding from lending institutions and other monetary sources; and helps to attract equity investment (4, 5).

Financial feasibility has been used more in developed countries. Fiedler et al. (6) examined the economic feasibility of maize flour and maize meal fortification in Kenya, Uganda, and Zambia and found that fortification is economically feasible, and would reduce deficiencies of multiple micronutrients, which are significant public health problems in each of these countries. Simon and Simon (7) found that Electronic Medical Records can provide both tangible (monetary) and intangible (clinical/quality of care) returns for the healthcare provider. Ohsfeldt et al. (8) undertook a financial feasibility and financial impact analysis of the implementation of Hospital Computerized Physician Order Entry (CPOE) systems and found that adoption of CPOE may be financially infeasible for these small hospitals in the absence of increases in hospital payments or ongoing subsidies from third parties. Morrison (9), examined the financial feasibility of robotics in neurorehabilitation and found that adding robotics to a hospital-based outpatient physical therapy clinic can be financially feasible. Bailit et al. (10) investigated the financial Feasibility of a Model School-Based Dental Program in different US States and found that the program is financially feasible and has considerable promise for reducing access disparities at a lower cost per child. Sabot et al. (11) examined the costs and financial feasibility of malaria elimination and found that the probability that elimination would be cost-saving over 50 years ranged from 0 to 42%, with only one site achieving cost-savings in the base case. These findings show that elimination might still be a worthy investment if total benefits are sufficient to outweigh marginal costs. Yarbro and Mehlenbeck (12) developed a financially feasible model that integrates behavioral health services into a pediatric endocrinology clinic, and concluded that a maximum of four patients per half day clinic was required to break even.

It is noted that financial feasibility analysis has commonly been used in non-health contexts (13). For instance, it has been used to model the economic feasibility of biomass delivery across fuel and product prices (13). In the study by Smith et al. (14), they found that a biodigester is not a financially feasible investment for a rural household but that substantial economic benefits are, however, found to make a biodigester a worthwhile investment from a broader societal perspective.

Financial feasibility analysis, though more prevalent in the developed countries, has been equally utilized by various governments and organizations in less developed economies to assess the viability of public health services before undertaking them. In 2008, the World Health Organization assessed the financial feasibility of a Social Health Insurance scheme in Swaziland and found that universal health coverage for all citizens was feasible as long as government funding was maintained and increased in line with inflation and GDP growth (15). Manjunath et al. (16) assessed the performance of 2 Social Health Insurance Schemes in India. They performed a comparative analysis of the cost structure, package rates and financial feasibility for 210 surgeries in a large hospital. Among other findings, the study revealed low financial viability of the hospital.

This paper presents evidence on the financial feasibility of using the BHCPF to provide a basic benefit package of maternal and child health services, which are considered the minimum set of services that such funds should provide. It also determined the unit costs of the MCH services in three states in Nigeria; and examined the potentials of additional funding options for extending coverage with a basic maternal and child health package.

## Methods

### Concept of financial feasibility

A financial feasibility assessment involves a comparison of all revenue sources to anticipated costs or expenditure, such that an excess of income over expenditure confirms viability. Hence, two components are required: “a costing of the service and an analysis of the revenue that will be used to provide the service at the stated costs. Financial feasibility is a study on whether a project is viable after taking into consideration its total costs and probable revenues. If the revenues cover the costs of the project, then the project is viable”^1^.

**Figure 1:**
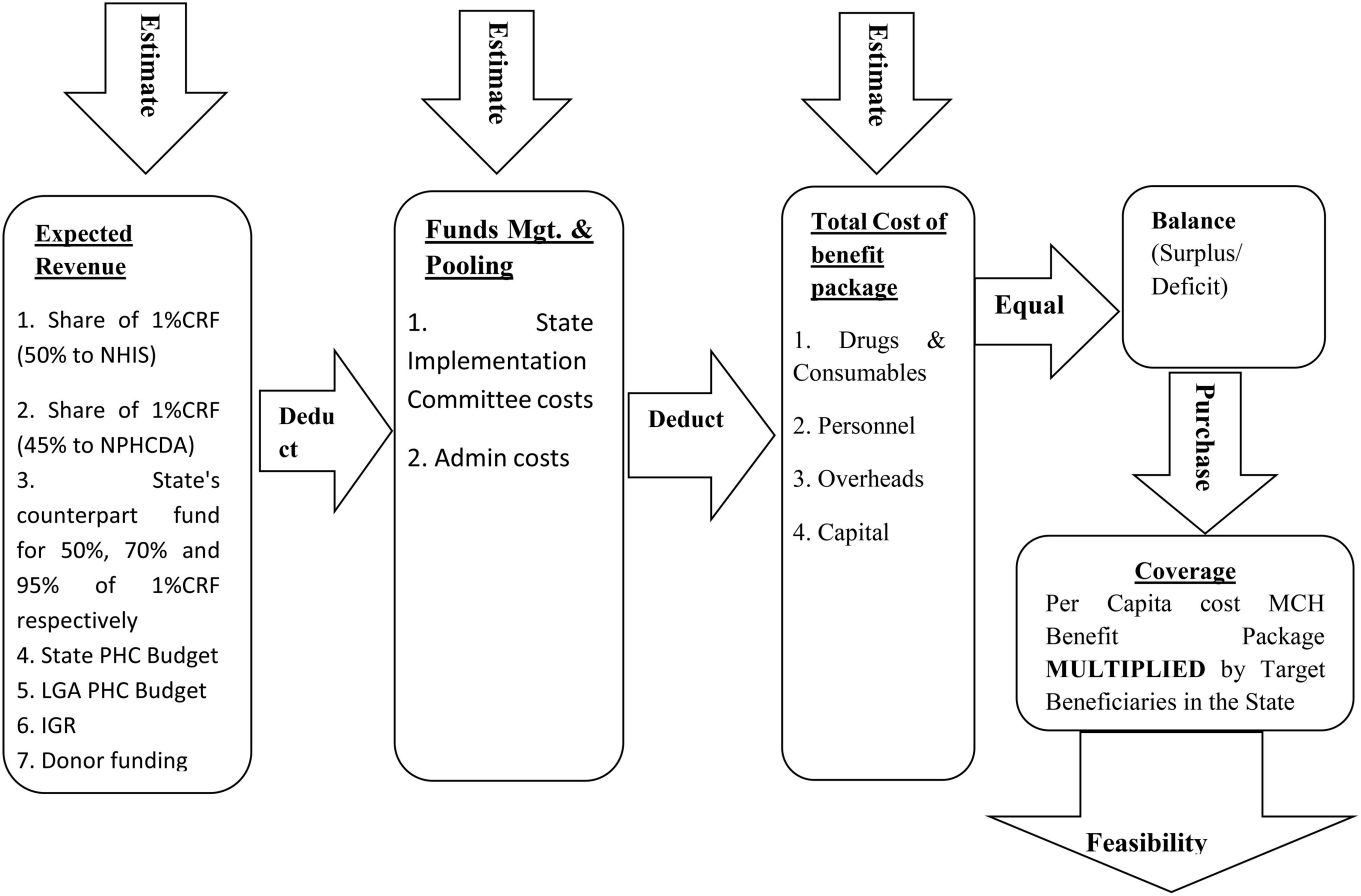
Steps in undertaking financial feasibility.

Assumptions may be made about different factors which could affect income sources and expenditures, and such assumptions can be varied to show different scenarios; the essence being to anticipate all possible outcomes. For example, the impact on administrative costs of utilizing middlemen or other intermediaries in the service delivery chain can be assessed by varying the fees paid to them or seeing what the expenditure profile looks like when these fees are excluded. The accuracy of the data used for a feasibility analysis determines how precise and reliable the output is. In order to perform the financial feasibility assessment therefore, two major components required are, (a) a costing of the MCH services to be provided and (b) an analysis of the revenue used to provide the services at the stated costs. These are illustrated in Figure 1.

### Study area

The study focused primarily on three states: Imo (southeast), Kaduna (northwest), and Niger (northcentral), Nigeria. In each state, two local government areas (LGAs)–one urban and one rural, were purposively chosen for facility and LGA level data collection. Within each state, two primary healthcare (PHC) facilities were visited in each of the two LGAs, to allow for the performance of financial feasibility based on different contexts.

Information was collected between April and June 2016 from staff of health facilities, local government councils, and from the state ministries of health. Key documents such as state and LGA budgets, Joint Annual Review Reports, State Strategic Health Development Plans, State HMIS reports and facility attendance, immunization, delivery, pharmacy and revenue registers were examined and relevant information collected from them.

### Data collection

The study team comprised health economists, public health specialists and financial analysts. Information was collected between April and June 2016 from staff of health facilities, local government councils, and from the state ministries of health. Key documents such as state and LGA budgets, Joint Annual Review Reports, State Strategic Health Development Plans, State HMIS reports and facility attendance, immunization, delivery, pharmacy and revenue registers were examined and relevant information collected from them. Cost data was collected at the PHC, LGA and State levels in the three states for the year 2015. Cost data included both costs of delivering MCH services (consumables and supplies, personnel, overheads, and capital costs), and administrative costs (e.g., State Implementation Committee, Health Maintenance Organizations (HMOs) administration fees and other administrative costs). Capital items at the facilities were categorized into building, transport, medical equipment, available at the facility as long as they were listed in the NPHCDA requirements for a standard PHC facility (17). Facility level costs were scaled up to the state level based on the estimated number of standard PHC facilities in the state. These were calculated to be 418 (Imo), 255 (Kaduna), and 274 (Niger).

Revenue data was collected at the PHC, LGA and State levels as well as at the National level for the year 2015. The revenue from the national level was the BHCPF. This amounted to about 38,555,000,000 Naira (1% of CRF). At the facility level, actual revenue data for 2015 were scaled up to the state level based on an estimate of the number of standard PHC facilities in each state. Other sources of revenue were State counterpart funding, State PHC budgets for each of the three states, and LGA PHC budgets as well as Internally Generated Revenue (IGR) from the PHC facilities in the States, which were scaled up to state level. Counterpart funding was computed as 25% of the NPHCDA (45%) component of the BHCPF.

### Data analysis

A basic benefit package, divided into maternal and child services was used for the purpose of the analysis. The services in the benefit package included antenatal care (ANC), delivery, post-natal care (PNC), family planning (FP), treatment of malaria, pneumonia, diarrhea, and routine immunization. Other routine services such as health education and growth promotion services, provided alongside earlier mentioned services, are assumed to be included in the personnel, capital and overhead expenditures for the services within the specific benefit package. The total service cost for drugs and consumables were based on the provision of MCH services constituting a standard benefit package for a pregnant woman and a child under 5 years. Where several visits are needed (e.g. for antenatal care) capital, personnel and overhead costs were allocated for each of the required minimum of four visits. The standard package of MCH services are to be purchased from PHC facilities in the states.

Tables 1–3 further summarize the specific methods applied for the determination of costs, revenue, benefits and financial feasibility. Table 1 presents the basis for deriving the data on demographics. This is in relationship to the target population, utilization weights for facilities and numbers of standard PHC facilities per state.

**Table 1:** Demographic figures.

**Subject**	**Description**
**DEMOGRAPHICS**	
Target population	Under 5-year-old Children (20%) and Pregnant Women (5%) out of a projected population of the state for 2015, based on 2006 Census figures and a growth rate of 3.2 (Imo), 3.0 (Kaduna), and 3.4 (Niger) *(Federal Republic of Nigeria: 2006 Population Census,)* http://www.nigerianstat.gov.ng
Utilization weights for facilities	Capital, personnel, and variable costs were allocated to MCH services based on the Utilization weights, determined as the proportion of all outpatient service users that attended for MCH services
Number of standard PHC facilities in the state	1 PHC per ward *(NPHCDA, Minimum Standards for Primary Health Care in Nigeria)*

Table 2 elaborates on the methods that were used to compute different costs and revenue. Costs were categorized into capital, overhead, personnel costs, and drugs & consumables. Revenues were estimated at the PHC, State and National levels. All the costs and revenues were in 2015 levels.

**Table 2:** Methods for deriving costs and revenue.

**Subject**	**Description**
**COSTS**	
Capital cost	The capital asset/items were annualized to allow for differential timing of capital assets. The share of the capital costs for MCH services was determined by adjusting the total costs by the facility utilization weight for MCH services. Unit costs were determined based on the patient visits for MCH.
Overhead costs	Total Annual expenditure on administration and overheads including travels and transport, utility, printing and stationery, maintenance, fuel and lubricant, staff training, and financial charges. The share of the overhead costs for MCH services was determined by adjusting the total costs by the facility utilization weight for MCH services. Unit costs were determined based on the patient visits for MCH.
Personnel costs	Total Annual expenditure on salaries of staff, including short term informal employees paid by the facility to fill gaps where these existed. The share of the personnel costs for MCH services was determined by adjusting the total costs by the facility utilization weight for MCH services. Unit costs were determined based on the patient visits for MCH.
Drugs and consumables	Cost of drugs and consumables for management of specific maternal and child conditions based on treatment standards prescribed by the SURE-P MCH services. *(USAID | DELIVER PROJECT, Task Order 4. 2014. Nigeria: 2014-2015 SURE-P Maternal and Child Health Commodity Requirements and Financing Needs. Arlington, VA.: USAID | DELIVER PROJECT, Task Order 4.)* Where different drugs (e.g., Amoxicillin and Co-trimoxazole) or commodities (e.g. IUCDs and Depo Provera) could be used, the costs were adjusted to reflect the probability of their use as reflected by the facility utilization records. A Uniform price list for drugs used was sourced from the Enugu State's drug revolving fund systems which was comprehensive and representative. Facility prices were not used because of inconsistences and over-inflation in some cases especially where the drugs were sourced from private pharmacies.
State level cost of services	Average cost of services per facility times the recommended number of standard PHC facilities in a state (1 PHC per ward).
**REVENUE**	
PHC facility revenue i.e. internally generated revenue (IGR)	Average IGR from MCH services provided in the facility and the monetary value of the stock of drugs available in the facility's drug revolving fund, scaled up to the state level based on the recommended number of standard PHC facilities in the state.
LGA revenue	Average PHC budget at LGA level, scaled up to state level based on the number of LGAs in the state.
Estimation of state level revenue from PHC level and LGA level	Average revenue per PHC times number of standard PHC facilities in a state, scaled up to the state level based on the recommended number of standard PHC facilities in the state.
National level revenue	1% of the Consolidated Revenue of the Federation distributed as specified in the National Health Act.
State's share of the BHCPF	BHCPF shared equally across 36 states and the Federal Capital Territory.

**Table 3:** Benefits and feasibility and gap analysis.

**Subject**	**Description**
**Benefit package**	
Unit cost of thin benefit package	**Maternal:** The amount that will be required per year to provide free maternal health services to a pregnant woman that uses formal ANC at an average of 4 times, has normal delivery in a health facility, and receives post-natal care and family planning service. This is weighted based on the utilization pattern of various maternal health services in facilities used. **Child:** The amount that will be required per year to provide a set of health services for an under-five year old child that visits the health facility three times a year. This is weighted based on the utilization pattern of various child health services in facilities used. **Either mother or child:** The amount required for either a mother or child which is the sum of: Unit maternal cost *times* probability of being a mother (0.2), and Unit child cost *times* probability of being a child (0.8), based on the demographic figures. State level costs are multiples of the unit costs.

### Financial feasibility analysis

A coverage and funding gap analysis is performed to show the impact of different levels of revenue when only drugs and consumables are provided under maternal and child care services. The standard costs of a thin benefit package for the different states are multiplied by the number of target beneficiaries to determine the amount required for the year. Only revenue components are varied in the different options to enable a sensitivity analysis of the adequacy of coverage for the target beneficiaries. Financial feasibility is determined by the excess or otherwise of revenue over costs. Finally, the additional funding requirement needed is computed to assist future planning.

So many scenarios were examined in the study. The different scenarios are based on different assumptions of the costs that will be incurred in the MCH program and the sources of revenue. Scenario one assumes that only costs of drug and consumables will be borne by the program, Scenario 2 is based on the funding of drugs, consumables, and overheads only, while Scenario 3 focuses on the funding of all cost items (i.e., drugs, consumables, overheads, personnel, and capital). All the costs and revenues were in 2015 levels.

However, the main feasibility analysis was the Scenario one, where it is assumed that only drugs and consumables are provided under maternal and child care services. The scenario also reflects the situation in the defunct NHIS-MDG Free MCH program. Table 4 shows the sub-scenarios under Scenario 1 that were analyzed. Scenarios 1a to 1c assume that there will not be any counterpart funding and all the funds will be from the 1%CRF. Scenarios 1d to 1f assume that there will be full counterpart funding from the states and LGAs.

**Table 4:** Feasibility and gap analysis under scenario 1.

**Overall approach**	**Revenue minus the total cost of defined package, analyzed for different scenarios of revenue and cost. Analysis was done for maternal and child target beneficiaries at the state level per year**.	
	**Cost**	**Revenue**
Scenario 1a	Drugs and Consumables only	0.5(1%CRF) = NHIS only
Scenario 1b	Drugs and Consumables only	0.7(1%CRF) * = 0.5: NHIS for basic package and 0.2: NPHCDA for drugs and consumables only*
Scenario 1c	Drugs and Consumables only	0.95(1%CRF) * = 0.5: NHIS for basic package and 0.45: NPHCDA*
Scenario 1d	Drugs and Consumables only	0.5(1%CRF) + 0.25[0.5(1%CRF)]* ^*^State's counterpart fund
Scenario 1e	Drugs and Consumables only	0.7(1%CRF) + 0.25[0.7(1%CRF)]* ^*^State's counterpart fund
Scenario 1f	Drugs and Consumables only	0.95(1%CRF) + 0.25[0.95(1%CRF)]* ^*^State's counterpart fund

### Vulnerable group analysis

An assumption was made that since 63% of Nigerians (NBS, 2010) live below the poverty line and that the BHCPF could be used to cover just this population. Hence, the free MCH benefit package that will be covered by the BHCPF will be used to cover just 61% of the potential beneficiaries.

## Results

### Demographics

The target MCH population figures for 2015 were 1,306,143 (Imo), 1,994,184 (Kaduna), 1,334,287 (Niger). The target number of health facilities were 418, 255, and 274 for Imo, Kaduna, and Niger states respectively. Based on the facility outpatient attendance records, the utilization weights for MCH services in facilities in each state were 0.83 (Imo), 0.85 (Kaduna), and 0.9 (Niger). The average utilization of maternal services in 2015 was 817 (Imo), 4,690 (Kaduna), and 2,623 (Niger). For child services, average utilization for this same period was 3,658 (Imo), 8,671 (Kaduna), and 5,555 (Niger).

### Cost analysis

The cost of delivering these services for 2015 were categorized into capital (i.e., building, transport, medical equipment, and others) costs, and recurrent (i.e., drugs & consumables, personnel, and overheads) costs. The total capital cost attributable to MCH services for the states for 2015 were − 248,852,530 Naira (Imo), 149,420,695 Naira (Kaduna), and 182,132,833 Naira. The total cost for providing drugs and consumables for 2015 at the PHC facilities were − 454,991,616 Naira (Imo), 913,922,222 Naira (Kaduna), and 925,283,827 Naira (Niger). The total personnel costs at the PHC facilities for 2015 were 149,889,054 (Imo), 2,304,646,474 Naira (Kaduna), and 3,916,955,570 Naira (Niger). Total overhead costs for the PHC facilities for 2015 were − 149,889,054 Naira (Imo), 32,910,644 Naira (Kaduna), and 44,514,106 Naira (Niger). The average unit capital costs for the states were 133.1 Naira (Imo), 43.9 Naira (Kaduna), and 81.3 Naira (Niger). Average unit personnel costs were 2,506.6 Naira (Imo), 676.5 Naira (Kaduna), and 1,748.0 Naira (Niger). Average unit overhead costs were 80.1 Naira (Imo), 9.7 Naira (Kaduna), and 19.9 Naira (Niger).

Table 5 shows the unit costs of providing drugs and consumables for various MCH services at a standard PHC facility in 2015. For example, the unit cost for antenatal care was 2,837.6 Naira, normal delivery was 1,503.2 Naira while malaria treatment costs 472.4 Naira per child.

**Table 5:** Unit costs for Drugs and Consumables for MCH services in the PHC facilities.

	**MCH Services**	**Unit cost of drugs and consumables in Naira (US$)**
Maternal	Antenatal Care	2,837.6 ($9.3)
	Normal Delivery	1,503.2 ($4.9)
	Postnatal care	339.8 ($1.1)
	Malaria in Pregnancy	423.5 ($1.4)
	Hypertension in Pregnancy	1,620.8 ($5.3)
	Postpartum hemorrhage	507.8 ($1.7)
	Family Planning	365.7 ($1.2)
Child	Treatment of Malaria	472.4 ($1.5)
	Treatment of Pneumonia	875.5 ($2.9)
	Treatment of diarrhea	443.1 ($1.5)
	Routine immunization	71.2 ($0.2)
	Measles	100.0 ($0.3)
	Neonatal Jaundice	–

*Prevailing exchange rate of 1US$ to 305 Naira*.

To provide a standard package of MCH services, it cost 21,145.5 Naira (Imo), 9,153.6 Naira (Kaduna), and 8,729.6 Naira (Niger) for a woman, and 8,544.8 Naira (Imo), 2,534.0 Naira (Kaduna), and 4,709.0 Naira (Niger) for a child in 2015.

### Revenue

Each state would receive 521,013,514 Naira and 468,912,162 Naira as shares of the BHCPF from the NHIS (50%) and NPHCDA (45%) components respectively. Counterpart funding for each state was also 24,481,419 Naira. Based on the different revenue sources earlier identified, the total revenue for each state is − 7,013,884,907 Naira (Imo), 9,968,254,995 Naira (Kaduna), and 5,202,936,421 Naira (Niger).

### Financial feasibility analysis

#### Scenario 1: only drugs and consumables are provided under MCH services

Table 6 shows that the entire available amount of the 1% CRF that is allocated to the NHIS (50%) will not be adequate to cover the benefit package for all the pregnant women in the three states. The highest gap was found in Kaduna State. However, the money will be enough to cover the benefit package for all under-five children in Imo state, but not in Kaduna and Niger states. Thus, for a comprehensive MCH benefit package, the funds will not be adequate to cover all the beneficiaries in all the states, with significant funding gaps.

**Table 6:** Scenario: 1a (Revenue: 50% of BHCPF).

**State**	**Unit cost of benefit package Naira (US$)**	**Target beneficiaries**	**Amount required/year Naira**	**Amount available in 2015 Naira**	**Lives covered**	**Gap/Surplus Naira**	**Additional aggregate fund needed(%)**
**MATERNAL**							
Imo	4,827 (15.8)	261,229	1,260,836,278	521,013,514	107,937	−739,822,765	142
Kaduna	4,774 (15.7)	398,837	1,903,905,766	521,013,514	109,144	−1,382,892,252	265
Niger	4,759 (15.6)	266,857	1,269,963,038	521,013,514	109,481	−748,949,524	144
**CHILD**							
Imo	385 (1.3)	1,044,915	402,574,841	521,013,514	1,352,332	118,438,672	−23
Kaduna	344 (1.1)	1,595,347	548,852,075	521,013,514	1,514,429	−27,838,562	5
Niger	843 (2.8)	1,067,430	900,339,922	521,013,514	617,706	−379,326,408	73
**ALL MCH**							
Imo	1,274 (4.2)	1,306,143	1,663,411,119	521,013,514	409,110	−1,142,397,606	219
Kaduna	1,230 (4.0)	1,994,184	2,452,757,841	521,013,514	423,603	−1,931,744,328	371
Niger	1,627 (5.3)	1,334,287	2,170,302,960	521,013,514	320,315	−1,649,289,446	317
**ALL MCH VULNERABLE GROUP**							
Imo	1,274 (4.2)	822,870	1,048,336,495	521,013,514	408,959	−527,322,981	101
Kaduna	1,230 (4.0)	1,256,336	1,545,293,182	521,013,514	423,588	−1,024,279,668	197
Niger	1,627 (5.3)	840,601	1,367,657,518	521,013,514	320,230	−846,644,044	162

Table 7 shows that if the 45% of BHCPF that is allocated to the NPHCDA is added to the 50% of the NHIS (95% BHCPF), the coverage improves and the funding gap significantly decreases. However, the entire available funds will still not be adequate to cover the benefit package for all the pregnant women in the three states. The highest gap was also found in Kaduna State. However, the money will be enough to cover the benefit package for all under-five children in the three states. Thus, for a comprehensive MCH benefit package, the funds will still not be adequate to cover all the beneficiaries in all the states, with significant funding gaps.

**Table 7:** Scenario: 1c (Revenue: 95% of BHCPF).

**State**	**Unit cost of benefit package Naira (US$)**	**Target beneficiaries**	**Amount required/year Naira**	**Amount available in 2015 Naira**	**Lives covered**	**Gap/Surplus Naira**	**Additional aggregate fund needed(%)**
**MATERNAL**							
Imo	4,827 (15.8)	261,229	1,260,836,278	989,925,676	205,100	−270,910,603	27
Kaduna	4,774 (15.7)	398,837	1,903,905,766	989,925,676	207,373	−913,980,090	92
Niger	4,759 (15.6)	266,857	1,269,963,038	989,925,676	208,013	−280,037,362	28
**CHILD**							
Imo	385 (1.3)	1,044,915	402,574,841	989,925,676	2,569,430	587,350,835	−59
Kaduna	344 (1.1)	1,595,347	548,852,075	989,925,676	2,877,414	441,073,601	−45
Niger	843 (2.8)	1,067,430	900,339,922	989,925,676	1,173,641	89,585,754	−9
**ALL MCH**							
Imo	1,274 (4.2)	1,306,143	1,663,411,119	989,925,676	777,309	−673,485,444	68
Kaduna	1,230 (4.0)	1,994,184	2,452,757,841	989,925,676	804,847	−1,462,832,165	148
Niger	1,627 (5.3)	1,334,287	2,170,302,960	989,925,676	608,599	−1,180,377,284	119
**ALL MCH VULNERABLE GROUP**							
Imo	1,274 (4.2)	822,870	1,048,336,495	989,925,676	777,022	−58,410,819	6
Kaduna	1,230 (4.0)	1,256,336	1,545,293,182	989,925,676	804,818	−555,367,506	56
Niger	1,627 (5.3)	840,601	1,367,657,518	989,925,676	608,436	−377,731,842	38

Table 8 shows that if the counterpart funding of 25% is added to the 50% of the NHIS, the coverage improves, but the funding gap remains. However, the entire available funds will still not be adequate to cover the benefit package for all the pregnant women in the three states. The highest gap was also found in Kaduna State. However, the money will be enough to cover the benefit package for all under-five children in Imo and Kaduna states, but not Niger state. Thus, for a comprehensive MCH benefit package, the funds will still not be adequate in all the states, with significant funding gaps.

**Table 8:** Scenario: 1d (Revenue: 50% of BHCPF plus counterpart fund (0.25 of 50%).

**State**	**Unit cost of benefit package Naira (US$)**	**Target beneficiaries**	**Amount required/year Naira**	**Amount available in 2015 Naira**	**Lives covered**	**Gap/Surplus Naira**	**Additional aggregate fund needed(%)**
**MATERNAL**							
Imo	4,827 (15.8)	261,229	1,260,836,278	651,266,892	134,934	−609,569,386	94
Kaduna	4,774 (15.7)	398,837	1,903,905,766	651,266,892	136,430	−1,252,638,874	192
Niger	4,759 (15.6)	266,857	1,269,963,038	651,266,892	136,851	−618,696,146	95
**CHILD**							
Imo	1,274 (4.2)	1,044,915	402,574,841	651,266,892	1,690,415	248,692,051	−38
Kaduna	1,230 (4.0)	1,595,347	548,852,075	651,266,892	1,893,036	102,414,817	−16
Niger	1,627 (5.3)	1,067,430	900,339,922	651,266,892	772,132	−249,073,030	38
**ALL MCH**							
Imo	1,274 (4.2)	1,306,143	1,663,411,119	651,266,892	511,388	−1,012,144,227	155
Kaduna	1,230 (4.0)	1,994,184	2,452,757,841	651,266,892	529,504	−1,801,490,949	277
Niger	1,627 (5.3)	1,334,287	2,170,302,960	651,266,892	400,394	−1,519,036,068	233
**ALL MCH VULNERABLE GROUP**							
Imo	1,274 (4.2)	822,870	1,048,336,495	651,266,892	511,198	−397,069,603	61
Kaduna	1,230 (4.0)	1,256,336	1,545,293,182	651,266,892	529,485	−894,026,290	137
Niger	1,627 (5.3)	840,601	1,367,657,518	651,266,892	400,287	−716,390,626	110

Table 9 shows that if the counterpart funding of 25% is added to the 50% of the NHIS and 45% of NPHCDA, there will still be funding gaps. However, the money will be enough to cover the benefit package for all under-five children in the three states, without any funding of maternal health services. Thus, for a comprehensive MCH benefit package, the funds will still not be adequate in all the states, with significant funding gaps.

**Table 9:** Scenario: 1f (Revenue: 95% of BHCPF plus counterpart fund (0.25 of 95%).

**State**	**Unit cost of benefit package Naira (US$)**	**Target beneficiaries**	**Amount required/year Naira**	**Amount available in 2015 Naira**	**Lives covered**	**Gap/Surplus Naira**	**Additional aggregate fund needed(%)**
**MATERNAL**							
Imo	4,827 (15.8)	261,229	1,260,836,278	1,237,407,095	256,374	−23,429,184	2
Kaduna	4,774 (15.7)	398,837	1,903,905,766	1,237,407,095	259,216	−666,498,671	54
Niger	4,759 (15.6)	266,857	1,269,963,038	1,237,407,095	260,016	−32,555,943	3
**CHILD**							
Imo	385 (1.3)	1,044,915	402,574,841	1,237,407,095	3,211,788	834,832,254	−67
Kaduna	344 (1.1)	1,595,347	548,852,075	1,237,407,095	3,596,768	688,555,020	−56
Niger	843 (2.8)	1,067,430	900,339,922	1,237,407,095	1,467,052	337,067,173	−27
**ALL MCH**							
Imo	1,274 (4.2)	1,306,143	1,663,411,119	1,237,407,095	971,637	−426,004,025	34
Kaduna	1,230 (4.0)	1,994,184	2,452,757,841	1,237,407,095	1,006,058	−1,215,350,746	98
Niger	1,627 (5.3)	1,334,287	2,170,302,960	1,237,407,095	760,749	−932,895,865	75
**ALL MCH VULNERABLE GROUP**							
Imo	1,274 (4.2)	822,870	1,048,336,495	1,237,407,095	971,277	189,070,600	−15
Kaduna	1,230 (4.0)	1,256,336	1,545,293,182	1,237,407,095	1,006,022	−307,886,087	25
Niger	1,627 (5.3)	840,601	1,367,657,518	1,237,407,095	760,545	−130,250,423	11

#### Scenario 2 and 3

An example of Scenario 2 is 2f (moderate and unlikely), which involves three types of costs (drugs, consumables, and overheads) and the revenue is 95% of BHCPF and state counterpart fund (0.25 of 95%BHCPF). In this case, all the revenue sources will not be able to cover the costs of maternal services in the three states, but will cover child health services in the three states. However, the revenue will not be able to cover a combination of MCH services in the three states.

An example of Scenario 3 is 3f (extreme and unlikely), which involves all the costs (drugs, consumables, overheads, personnel, and capital) and the revenue is 95% of BHCPF and state counterpart fund (0.25 of 95%BHCPF) plus state and LGA budgets and internally generated revenue. In this case, all the revenue sources will be able to cover maternal services in the three states, child health services in Kaduna and Niger states and a combination of MCH services in only Kaduna state.

## Discussion

The findings show that allocating just 1% of consolidated revenue fund of the federation as the BHCPF will not be enough to assure universal financial protection of a basic health benefit package needs of all pregnant women and children under five in the states based on the most optimistic and realistic Scenario 1. The various funding scenarios that were examined show the inadequacy of available funds to meet the needs of the target beneficiaries. The only feasible options are funding of child services only utilizing at least 70% of the CRF in addition to other revenue sources with counterpart funding from the states and local governments. It is also feasible to cover a limited percentage of vulnerable pregnant women and under-five children. Hence budgetary constraints will constrain the achievement of UHC for MCH based solely on the BHCPF. This is similar to findings in another context in by Hendriks et al. (18) who argued that national health budget considerations in low and middle income countries might lead decision-makers to choose other investments with higher health impact for a budget equivalent to certain high priority health interventions such as vaccines.

The available funds from the BHCPF should be used immediately to cover the maximum numbers of mothers and children that it can, whilst sourcing for additional funds to ensure universal coverage of services. The available funds could for instance be used to target high priority population groups such as the like the poor and high risks pregnancies, whilst the state governments source for additional funds to cover all the targeted beneficiaries. The states and LGAs should also budget and pay counterpart funding for the BHCPF to cover an appreciable numbers of the target beneficiaries. The findings could also serve as a basis for incentivizing beneficiaries to utilize the services as was fund in a study in Pakistan that was used to provide information on a cost package for services (19).

The study highlights the importance of undertaking a financial feasibility analysis to provide the evidence-base of what MCH services and population groups that the BHCPF can realistically cover. It is noted that financial feasibility analysis is now being used in different contexts in the health sector (20). The methodology was used in sub-Saharan Africa (SSA) in a study by van den Heeveri (21) that examined the financial feasibility review of National Health Insurance (NHI) proposals for South Africa and found that there is little evidence to support the central objective of a reform which seeks to raise up to 5% of Gross Domestic Product (GDP) in additional taxes to achieve a total public spend equivalent to 8% of GDP. In the study by Witter et al. (22) that examined the financial feasibility or value for money for the Sierra Leone Free Health Care Initiative (FHCI), it was deemed to provide value for money although the results on the extent of the program's financial feasibility was not provided.

The funding gap analysis shows that states would require more funds in order to provide MCH services to target beneficiaries. However, it will not be feasible to devote all the state health revenue (Scenario 3) to only MCH services. More than half of those who need these services cannot access them in the States. Even when other cost components such as overheads and capital are removed from the benefit package, available funds from the BHCPF and State counterpart are inadequate to meet the required level of coverage. Personnel costs constituted the largest cost component. The results from a study in Tajikistan (23) was used for a purpose that is similar to the current study because it was used to determine the level of coverage and benefits that will be derived from a hypothetical investment of $20 million in MCH services.

It could be argued that the unit costs of the services could be smaller if the status quo is used to cover for capital and personnel costs, whilst the BHCPF is used to provide only commodities. However, a counter argument is that the health facilities rarely receive overhead budgets from the government and the BHCPF is expected to bridge the gap in funding for overheads budgets, which are needed to effectively run the health facilities. Also, many health facilities currently have insufficient staff complement when compared to the minimum standards specified by the NPHCDA. The basic benefit package for both mothers and children provides an initial minimum package to which further services could be added as funding increases. Hence, in order to meet the stated target of providing units of a basic benefit package and meet up with the minimum standards of health staff, additional funds from the BHCPF are also needed to engage more staff.

The BHCPF is potentially a good step toward providing affordable healthcare for pregnant women and children under five years, but the current level of funding will not assure UHC for all the target beneficiaries. This highlights the importance of undertaking a financial feasibility analysis before program implementation so as to be aware of what the funds can cover (24). In a study by Hecht et al. (25), they found that the proposed intervention on hepatitis B and C vaccines can be accommodated within South Africa's fiscal space and represents good use of scarce resources and that their work illustrates the value and feasibility of using an investment case approach to assess the costs and relative priority of scaling up health services.

In recognition that BHCPF is insufficient to fund services for the target group. There is a need to re-evaluate the level of funds to be allocated as BHCPF and the analysis shows that at least 4% of CRF is the minimum that will cover the all target beneficiaries within the state based on just 50% of the BHCPF to the NHIS. Counterpart funding must be entrenched in the design and implementation of the program, so as to ensure that more beneficiaries are covered. There is also the need to explore funding from different sources such as from budget allocations, development partners, health insurance schemes at the national, state, and community levels as well as internally generated revenue at state and LGA levels. For instance, basket funds can be utilized to increase funding from the state and local government level. Meghani et al. (26) describes basket funds as a means of pooling funds from different sources such as governments, donor agencies, and the private sector to achieve defined objectives, in this case, primary health care services. This arrangement has been implemented in Zamfara and Kano states with great success. However, establishing a basket fund requires the strong support of key actors at the state and local government levels to ensure its success.

The study limitations are related to the major challenges in data collection. There were significant variations in the costs of drugs at the facility levels and costs indicated at the state Central Medical Stores for drugs used in the facilities. In some cases, there was reluctance to provide the actual costs/prices of drugs. A uniform price list obtained from the functional Central Medical Store in Enugu State which was closest to the actual market prices of drugs was used for all computations. Also, there are several shortcomings with data summarization in the health facilities and the state HMIS system. In addition, it was not possible to obtain data on actual utilization of the NHIS/MDG programme from any of the parties involved (NHIS, Providers, State Implementation Committees, and HMOs). The accountability mechanisms and reporting systems for any future programme must be well laid out and appropriate sanctions for defaults clearly specified.

The State Ministries of Health should be encouraged to utilize a similar feasibility analysis tool for budget negotiations. This would show in clear and quantitative terms the potential impact of revenue and expenditure decisions on health sector objectives and engender policy level support for improved budgetary allocation to the sector. Finally, in order to increase coverage with some services, the BHCPF could be channeled to provide only drugs and consumables, with states expected to continue their funding of other cost elements such as personnel and overhead costs. However, adequate steps should be taken to ensure that personnel and overheads are sufficiently provided by states.

## Ethics statement

This study was carried out in accordance with the recommendations of the guidelines of the University of Nigeria research policy and the University of Nigeria Senate Research Grants Committee. The protocol was approved by the University of Nigeria teaching Hospital Ethics Committee. All subjects gave written informed consent in accordance with the Declaration of Helsinki.

## Author contributions

OO and HW conceptualized the study; OO, CO, IN, HI, and BU collected the data; OO, CO, HW, and HI undertook the data analysis; OO drafted the first draft of the paper, which was revised by all the co-authors. All the co-authors approved the final draft of the paper.

### Conflict of interest statement

The authors declare that the research was conducted in the absence of any commercial or financial relationships that could be construed as a potential conflict of interest.

## Footnotes

^1^http://www.investorwords.com/15399/financial_feasibility.html#ixzz4kJIaeNQ0
